# Knowledge of India as an Instrument of Nazi Politics: Ludwig Alsdorf, German Indology and Indian Anti-Colonialism

**DOI:** 10.1007/s00048-023-00364-z

**Published:** 2023-08-02

**Authors:** Baijayanti Roy

**Affiliations:** Goethe-Universität Frankfurt, Frankfurt, Germany

**Keywords:** Nazism, India, Indology, Anti-colonialism, Ludwig Alsdorf, Subhas Chandra Bose

## Abstract

Ludwig Alsdorf (1904–1978) is primarily remembered as a scholar of ancient and medieval India. This paper examines a little known aspect of Alsdorf’s career: his role as an expert of modern India in Nazi Germany. Alsdorf, who was in India from 1930 to 1932, joined the NSDAP and a few of its subsidiaries after 1933. Political contacts as well as his claims of having “first-hand experience” of India secured Alsdorf writing assignments that aimed to fulfil the regime’s political objectives. In return, he gained professional advancement and the reputation of being an authority on modern India. This paper reviews Alsdorf’s trajectory within the NS state by focussing on the following aspects: the ways in which Alsdorf offered his knowledge of India to the Nazi regime; the material and symbolic resources that he received in return; the relative importance of political affiliations, professional networks and academic accomplishments for Alsdorf’s career; the “politics of the past” practised by Alsdorf and some of peers after 1945; and the (re)presentation of the “uses” of Indology in the “Third Reich” and in the Federal Republic of Germany by Alsdorf and his colleagues.

## Abstract (de)

Ludwig Alsdorf (1904–1978) wird vornehmlich als Gelehrter des Jainismus erinnert. Dieser Aufsatz betrachtet einen weniger bekannten Aspekt seiner Karriere: seine Rolle als Experte des modernen Indiens im nationalsozialistischen Deutschland. Alsdorf, der 1930 bis 1932 in Indien war, trat 1933 der NSDAP und einigen ihrer Unterorganisationen bei. Sowohl seine politischen Kontakte als auch seine Behauptung, Indien „aus erster Hand“ zu kennen, sicherten ihm Schreibaufträge, die den politischen Zielen des Regimes dienten. Im Gegenzug konnte Alsdorf eine Karriere sowie den Ruf als Autorität für das moderne Indien aufbauen. Dieser Aufsatz betrachtet Alsdorfs Werdegang im NS-Staat neu, indem er die folgenden Aspekte fokussiert: (1) die Art und Weise, wie Alsdorf dem NS-Regime sein Wissen anbot, (2) die materiellen und symbolischen Ressourcen, die er im Gegenzug erhielt, (3) die vergangenheitspolitischen Strategien, die Alsdorf und einige seiner Kollegen nach 1945 einsetzten und (4) die Art und Weise, wie Alsdorf und seine Kollegen, den „Nutzen“ von Indologie im „Dritten Reich“ und in Westdeutschland (re)präsentierten.

The Indologist Ludwig Alsdorf (1904–1978) is remembered for his valuable contribution to the study of Indian civilization in general and to Jainism, Buddhism and the Vedas in particular (Bruhn et al. 1990: vii). This article aims to examine a relatively obscure aspect of his career, namely, how Alsdorf, a promising researcher of medieval Jain texts, was temporarily transformed into an “expert” on modern India who provided the political knowledge required to fulfil the Nazi state’s policy towards contemporary India. This article maintains that the Nazi political authorities valued Alsdorf’s “expertise” on modern India much more than his brilliance in textual criticism or even his affiliation with certain Nazi organizations including the NSDAP. Such a perspective was in keeping with the Nazi regime’s general attitude towards intellectuals and academics. Alsdorf’s political utility, manifested through the strategic deployment of his knowledge of modern India, was rewarded by various centres of power in the National Socialist state with improved professional prospects and greater influence. Finally, this article looks at the “politics of the past” which Alsdorf engaged in. This entailed a retrospective refashioning of his trajectory during the Nazi period, which helped his rehabilitation in the post-war West German academia. A concomitant issue which this article examines is how Indology was presented by Alsdorf and others as an academic discipline (*Wissenschaft*) with political and practical relevance in the “Third Reich” and subsequently in the Federal Republic of Germany. This article thus contributes to the study of the complicated relationship between scholarship and politics during and beyond the years of Nazi dictatorship.

## Early Career and Nazi Associations

Ludwig Alsdorf, the son of an evangelical pastor from the Rhineland, studied Indian philology (mainly Sanskrit) as well as *Islamkunde* (Islamic languages and other subjects) at the University of Hamburg, where he completed his PhD in 1928 under Walther Schubring, professor of Indology and an expert on Jainism. Alsdorf’s dissertation was on a medieval Jain text, *Kumarapalapratibodha*, which included derivative dialects of Sanskrit (*apabhramsa*).

Alsdorf subsequently began to work under the Indologist Heinrich Lüders at the University of Berlin. With financial assistance from several governmental organizations including the Foreign Ministry, Alsdorf went to India on a study tour in October 1930. He taught German and French at the University of Allahabad in north India and used this opportunity to collect material for further research. He also travelled widely in India, as well as in Burma (now Myanmar) and Sri Lanka. This “first-hand experience” of the subcontinent would later be the foundation of Alsdorf’s special claim to being an expert on contemporary India.

In July 1932, Alsdorf returned to Germany. The ambitious young scholar lost little time in aligning himself with the prevailing political wind. He joined the Nazi party on 1 August 1933 (membership number 2697931).^1^ Alsdorf underwent compulsory labour service (*Arbeitsdienst*) in Bredtstadt from 17 January to 31 March 1934. This was followed by a three-week course at the Prussian Lecturers’ Academy (*Dozentenakademie*) connected to the University of Kiel. Lecturers’ Academies were training camps for those aspiring to be University professors. Here, scholars underwent physical drills as well as ideological indoctrination in order to fulfil the National Socialist ideal of a “scholar-soldier” who would serve the “Reich” at the University as well as on the battlefield. The Lecturers’ Academy that Alsdorf attended had the reputation of being a model centre for Nazi indoctrination (Göllnitz 2016: 54). Here, visiting scholars were graded in categories such as “National Socialist thinking” and “National Socialist disposition” along with physical fitness.^2^ A questionnaire answered by Alsdorf during this training reveals that his “special cultural political interests” were *Rassenkunde* (race science) and *Auslandsdeutschtum* (Germans outside Germany), subjects which enjoyed political currency in Nazi Germany.^3^

Alsdorf completed his *habilitation* (the second thesis required by German universities for professorships) in June 1935 from the University of Berlin. He was subsequently appointed as a lecturer for Indology there.^4^ His *habilitation* was an annotated translation of *Harivamshapurana*, a Jain narrative text written in *apabhramsa* by the poet Pushpadanta in the tenth century. Alsdorf’s dissertation had been well received in Indological circles. His *habilitation*, published in 1936, was praised not only by his academic peers, but also by Wolfgang Erxleben, who “evaluated” scholars and their works on behalf of the Nazi Party’s Department of “Sciences” (*Hauptamt Wissenschaft*) under Alfred Rosenberg. Erxleben considered it to be a “perfect example of textual criticism.”^5^ These two works established the young scholar’s reputation as one of the most gifted German Indologists of his generation.

Along with his academic pursuits, Alsdorf became involved in different organizations affiliated to the Nazi Party. He belonged to the Foreign Affairs section of the *Deutsche Dozentenschaft*, an association of non-tenured teaching staff at German universities.^6^ Membership in this thoroughly Nazified organization, which wielded some power in the universities in the initial years of the dictatorship, was compulsory for non-tenured lecturers (Nagel 2008: 118–119). Alsdorf’s association with the Foreign Affairs section indicates that he was building up his credentials as a specialist in the subject. In December 1936, Alsdorf joined the Nazi paramilitary organization, the National Socialist Motor Corps (*Nationalsozialistisches Kraftfreikorps *or NSKK) in which he seems to have progressed up the ranks.^7^ In a letter to the University of Münster on 18th February, 1938, justifying his candidature for a teaching post, Alsdorf proclaimed that he “indeed” belonged to the motorboat section (*Kraftbootsturm 4\Kb*) as a Second Lieutenant (*Sturmmann*) of the NSKK. By 1936, he had also joined the National Socialist Lecturers’ Association (NSDB).^8^

## Politically Unreliable?

Despite his involvement with these organizations, Alsdorf’s “political reliability”—a crucial yardstick for the Nazi state—seemed to have been a matter of dispute in the initial years of Nazi rule. Alsdorf received a positive review for his compulsory labour service. The certificate, signed by the divisional head and Field master Oldweiler, claimed that during his stay Alsdorf had “conducted himself well and had adjusted fully to the life in the camp” and that “he had, in an exemplary way, proved himself to be a true comrade.”^9^ A report from the NSDAP’s branch in Saarland, Alsdorf’s home state, applauded his commitment to the “German cause” in the so-called “battle for Saarland”—the successful Nazi propaganda campaign to “win” back the Saarland (Mühlen 1979).^10^ However, the report on Alsdorf provided by the Lecturers’ Academy at Kiel was unequivocally negative. The unsigned report sent to the University of Berlin claimed that Alsdorf was “politically opaque” and that his attitude could hardly be characterized as that of a National Socialist, not even as a Nazi academic. He was also “pompous and talkative” and “deeply egocentric.”^11^

Misgivings about Alsdorf’s commitment to National Socialism seems to have led to a debate within the Ministry of Education (*Reichs und Preußisches Ministerium für Wissenschaft, Erziehung und Volksbildung*) about whether he deserved to be given a teaching licence (*Venia Legendi*), which is normally awarded to those who successfully complete a *habilitation*. The existence of such a discussion is indicated in an untitled note found in Alsdorf’s file maintained by the Ministry of Education in Berlin. The note, dated 18 October, does not mention the year. It was written by a certain “HZ,” ostensibly a fellow Indologist who had accompanied Alsdorf to the International Orientalists’ Conference in Rome in 1935. HZ had kept watch over Alsdorf, who had reportedly presented a “noteworthy” (*beachtlich*) paper. It is likely that HZ was Heinrich Zimmer, the famous Indologist affiliated to the University of Heidelberg. HZ’s report stated that academically Alsdorf was “very good” but “it does not appear likely to me that he could be termed a National Socialist.” However, HZ expressed the hope that Alsdorf could be trained into becoming a committed National Socialist after the imminent appointment of the Indologist Bernhard Breloer, an ardent Nazi and SS functionary, as professor at the University of Berlin (Framke 2014: 94–95). The report also suggested that Alsdorf’s ideological indoctrination could be “rounded off” through military service. HZ concluded that Alsdorf should receive his teaching licence, which could be revoked later if necessary.^12^

Alsdorf did indeed receive his teaching licence and never provided the Nazi authorities any cause to withdraw it. Ironically, it was Heinrich Zimmer who, despite his efforts to conform to certain aspects of Nazi politics, was forced to forfeit his teaching licence and leave the country (Roy 2022a: 11–29). The note about Alsdorf indicates that Zimmer’s efforts to ingratiate himself with the Nazi regime did attain a modicum of success, but they were not enough to prevent his exodus.

It is difficult to ascertain whether and to what extent Breloer could “train” his younger colleague, since he provided divergent evaluations of Alsdorf. In 1936, Breloer wrote a letter to the dean of the Philosophical Faculty at the University of Berlin, requesting a raise for Alsdorf, who received a stipend instead of a salary. As justification, Breloer mentioned Alsdorf’s membership in the NSDB as well as his promising academic career.^13^ Breloer also wrote a letter of recommendation for Alsdorf to the dean of the Philosophical Faculty at the University of Münster in connection with a teaching post for which Alsdorf was being considered. In this letter, dated 14 April 1938, Breloer mentioned Alsdorf’s academic abilities and his helpfulness but not his political activities.^14^ In another, presumably confidential note without an addressee, written on 3 April 1938, Breloer praised Alsdorf’s academic works as well as his fluency in English and Hindustani. He also attested to Alsdorf’s Nazi affiliations, claiming that Alsdorf was a member of the Marine SA, the motorized water transport unit of the *Sturmabteilung *(SA), the notorious Nazi paramilitary outfit, as well as the Foreign Affairs section of the *Deutsche Dozentenschaft*. Since membership in the latter was compulsory for non-tenured lecturers like Alsdorf, the fact that Breloer mentioned that he belonged to the Foreign Affairs section indicates that this specialized function had political significance. Nevertheless, Breloer added the caveat that Alsdorf “had not addressed any ideological issues in his area of academic expertise so far.”^15^

 Breloer’s claim that Alsdorf had joined the Marine SA requires our attention, since it appears in no other official document. In the questionnaire for the Lecturers’ Academy mentioned earlier, Alsdorf denied being a member of either the SA or the SS.^16^

The Marine SA, based primarily in the port city of Hamburg, indulged in repeated bloodbaths to stamp out all political opposition, as a contemporary account indicates (Ehrenreich 1935). While the Marine SA and the motorboat section of the NSKK were officially separate entities, the border between the two was porous. It is probable that Breloer simply confused the two entities, or that Alsdorf, who lived in Hamburg until the spring of 1935, had “concluded his initial training” with the Marine SA (as Breloer wrote in the secret report) before joining the NSKK in 1936. Whether a member of the SA or not, Alsdorf’s membership in the NSKK indicates a firm commitment to Nazi ideology. The NSKK operated closely with the more visible and more notorious SA and SS, but unlike these two paramilitary organizations, it was not declared a criminal organization by the Allies after the war. Hence, after 1945, many former members of the NSKK could present their membership in this organization as the “lesser evil” (Hochstretter 2005). Alsdorf, as we shall see, also took recourse to this strategy after the war.

By April 1936, the NSDB at the University of Berlin became somewhat more optimistic about Breloer exerting a “positive” influence on Alsdorf. An unnamed representative of the NSDB wrote to Willi Willing, professor at the Technical University of Berlin and head of the NSDB of the *Gau* (administrative unit) of Berlin, that Alsdorf, who was working with Breloer “was readier than before to prove his political commitment,” although “no conclusive evaluation of his worldview could be provided yet.”^17^ Nevertheless, in 1938, Alsdorf unsuccessfully applied for the position of professor of Indian Philology at the University of Leipzig, which went to Friedrich Weller, an ex-student of the university. Weller was one of the signatories to the oath of allegiance that professors at German universities pledged to Adolf Hitler in November 1933 (Pollock 1993: 94). Loyalty to the National Socialist state was certainly an important factor in this appointment, as positive evaluations of Weller’s commitment to National Socialism, provided by various functionaries connected to the University of Leipzig, indicate.^18^ In contrast, a number of reports on Alsdorf from the secret service of the Nazi party (*Sicherheitsdienst* or SD) to the Ministry of Education of Sachsen cast doubts on his ideological integrity. One of these reports, dated 6 February 1937, which bears the illegible signature of the head of the SD subdivision (*Unterabschnitt*) of Dresden-Bautzen, stated that although Alsdorf’s academic record was undoubtedly first-rate, his political orientation remained uncertain. The report further claimed that Alsdorf was indifferent towards National Socialism, although he routinely contributed to different Nazi welfare schemes and signed his letters with “Heil Hitler.”^19^ Another report, dated 23 March 1937, unsigned and bearing the stamp of the head of the same SD subdivision, provided the damning verdict that “even though he is a party comrade, he associates less with National Socialist circles than with liberals at the University. In character, he appears almost like a climber (*streber*), who tries to use every situation to his own advantage.”^20^ The different assessments of Alsdorf were summed up in a report sent by Erhard Landt, assistant professor of Physics at the University of Berlin, on behalf of the NSDB to the Rector of the University on 24 March 1938. Landt, who was known as “one of the most fanatic supporters of National Socialism at the University” (von Lösch 1999: 253) repeated the allegations about Alsdorf’s dubious commitment to Nazism and that he appeared to be a self-seeking careerist. He concluded that Alsdorf was known to be “an unpleasant type of lecturer, who has not understood the political duties of the Universities.”^21^ This last sentence is important because it underscores the fact that despite his Nazi affiliations, Alsdorf at this point could not convince the relevant authorities that he corresponded to the ideal academic envisaged by the Nazi political establishment. Thus, the centres of power did not invest substantially in his career.

Alsdorf did obtain the post of lecturer and head of Indology at the University of Münster in October 1938, after the retirement of the Indologist Richard Schmidt who held a professorial chair there.^22^ The initiative to appoint Alsdorf seemed to have come from the University of Münster, as a letter from March 1938, sent by the dean of the Faculty of Philosophy to the “Reich” Education Minister Bernhard Rust, indicates.^23^ Although Alsdorf was chosen as Schmidt’s successor, his position did not come with tenure or a proper salary. The aforementioned letter justifying Alsdorf’s appointment also pointed out that the young Indologist from Berlin would continue to receive his stipend, thus generating no additional expenses for the University of Münster. The documents related to Alsdorf’s appointment, preserved at the archive of the University of Münster, suggest that University authorities did not overtly emphasize Alsdorf’s commitment to Nazi politics in the appointment process. This was probably due to the particular political path chosen by the University after 1933, wherein the academic staff adjusted to the new regime by making the courses they offered “politically relevant” rather than taking part in political activities (Thamer et al. 2012). This would also explain why Breloer omitted all references to Alsdorf’s involvement in the Nazi party and the NSKK in his recommendation letter to the University of Münster (dated 14 April 1938). Breloer probably considered such details irrelevant.

## Uses of Indology

Alsdorf’s appointment at the University of Münster provides a glimpse into the perceived “utility” of Indology to the Nazi state. These perceptions merit a closer look, since they offer a window into the complex entanglement of political and academic rationales validating the pursuit of Indology as an academic discipline in the “Third Reich.” The linguist Erich Hoffmann, professor for Comparative Philology and head of the Nazi Lecturers’ Association at the University of Münster, pleaded for Alsdorf’s appointment in a letter to the dean of the Faculty of Philosophy, dated 9 February 1938, claiming: “I consider it to be urgently necessary that Aryan Philology is reintroduced at the University of Münster. Serious philological research is impossible without an initiation into Sanskrit. The issue is not only about purely linguistic questions, but also about the problem of the original home of Indo-Germans which enjoys national political significance today.”^24^ For Hoffmann, Indology was synonymous with the study of Aryanism and the question of the “original homeland” of the Aryan people, which provided political capital to the Nazi regime. For Franz Taeschner, professor and director of Oriental Studies at the University of Münster and an expert on Islam, the usefulness of Indology was related primarily to Germany’s prestige in the world. Taeschner had entered the NSDAP in 1933 and provided his knowledge of Arabic and the Islamic world in different ways to the Nazi regime (Ellinger 2006: 366–367). In a letter to the dean, dated 15 February 1938, Taeschner claimed that ever since it was discovered that the Indians were Aryans and therefore related to the Germans, Indology has generated a deep respect for German culture among the Indian people. By cultivating Indology, German universities were thus fostering the friendship between Germans and Indians, which was immensely important for the “worldwide recognition” (*Weltgeltung*) of the Germans.^25^

Although several notions about the “usefulness” of Indology for the University of Münster as well as for the Nazi state can be gleaned from these accounts, it is far less clear why Alsdorf was thought to be a suitable candidate to succeed Richard Schmidt, who worked on lexicographic and cultural-historical works in Sanskrit. Politically however, there was an element of continuity since Schmidt, like Alsdorf, had joined the Nazi party in 1933.^26^ Both Alsdorf and Schmidt therefore conformed to the general political orientation of the University of Münster, where ninety percent of the professors were members of the NSDAP, though not all of them were involved in the party’s activities (Benz 2013). In this respect, Alsdorf’s political affiliation may not have been completely irrelevant to his appointment.

## 1938: A Watershed Year

Alsdorf took the “Hitler oath” (*Führereid*) on 12 December 1938, as required by the Ministry of Education.^27^ All government officials (*Beamte*) took this oath at the time of assuming office, pledging to remain loyal to the “Führer” of the German and Reich and people, Adolf Hitler. Alsdorf reiterated this oath in September 1939 when he was made an untenured government official with a proper salary replacing his stipend.^28^ The year 1938 was a watershed in Alsdorf’s career. Apart from his move to Münster, it was in 1938, according to Alsdorf’s statement after the war, that the historian Egmont Zechlin requested him to write a book about India for a series called *Weltpolitische Bücherei* (Library of World Politics).^29^ This book would spectacularly advance Alsdorf’s career in Nazi Germany. In his post-war statement to the University of Munich, Alsdorf claimed that Professor Zechlin assured him that this book would be purely academic; it would not be used for political purposes. Even a cursory look into Egmont Zechlin’s career casts doubt on this statement. Zechlin joined the NSDAP and the SA in 1933 (Eckert 2010: 91). His public lectures and writings during this period reflect his concurrence with many aspects of the Nazi *Weltanschauung* (Frees 2004: 338–339).

In 1940, Alfred Rosenberg, the head of the External Affairs department of the NSDAP, officially entrusted Zechlin and another loyal follower, Georg Leibbrandt, to co-edit the *Weltpolitischer Bücherei*. This series published books on different countries written by reputed scholars to “educate” the German people in world political affairs so that they could understand their “historical tasks,” as an article in the Nazi mouthpiece *Nationalsozialistische Monatshefte* claimed (Rudiger 1941: 74–75). It is likely that Alsdorf was chosen since he had demonstrated his knowledge of modern India through his work for the Foreign Affairs department of the *Deutsche Dozentenschaft*. Alsdorf himself hinted at the political importance of his book project in a letter that he wrote to the University authorities of Münster in February 1940, asking for leave to finish the book and mentioning that it had been commissioned by the External Affairs department of the NSDAP.^30^ The University also noted that Alsdorf was exempted from military duties until 30 June in order to complete his book.^31^

From 1939, the University of Münster also deployed Alsdorf’s knowledge of modern India for teaching courses. As Germany’s foreign policy turned increasingly belligerent, the University of Münster attempted to contribute to the country’s war-preparedness by introducing a course on “Knowledge of Foreign Countries and Colonies.” In the summer semester of 1939, Alsdorf lectured on “the great religions of India” as a part of this course. By the summer semester of 1940, he was offering courses in two “living” Indian languages—Hindustani and Gujarati—along with Sanskrit. He also lectured on the politically significant subject of “English rule and nationalist movement in India.”^32^ Alsdorf’s lectures on contemporary India reflected the shift in Nazi Germany’s policy towards India after Joachim von Ribbentrop became the foreign minister in 1938. Hitler admired the British Empire and wanted to build an alliance with it. However, as war with England loomed large, Ribbentrop and several other members of the Nazi ruling elite began to perceive the propagandistic importance of cultivating a positive image of Germany in India (Kuhlmann 2003: 69). This could be done best by criticizing British colonial rule and encouraging the Indian anti-colonial movement. For this, knowledge of contemporary India was necessary, which the University of Münster aimed to impart in order to prove its political relevance to the Nazi state.

## *Indien*

Alsdorf’s book for the Weltpolitische Bücherei, entitled *Indien* (India) was published in 1940. It was also the product of the change in Germany’s policy towards India. In order to conduct anti-British propaganda concerning India, it was necessary for the German political establishment to have discursive knowledge of the British colonization of India and the Indian anti-colonial movement. *Indien* provided this knowledge in an erudite but accessible style. The book devoted only one chapter to the history of India before British domination. Alsdorf used the following four chapters to elaborate on British rule in the subcontinent by focussing on the conquest of India by the British, the nature of British rule in India, the ways in which the British government guarded and defended its Indian territory and the rise of the Indian nationalist movement. The book critiqued British colonial rule in India and encouraged the Indian anti-colonial movement in a scholarly tone. *Indien* had a run of several editions. It won acclaim from the “Führer” himself, who, according to the National Socialist Lecturers’ Association, directed all high party functionaries to read it.^33^

What was special about *Indien*? It was not the first work written by a German academic expert on India that examined the history and effects of British colonialization. The Orientalist Josef Horovitz, for example, had written an influential book on the subject in 1928 (Horovitz 1928). But unlike the book by Horovitz, *Indien* indicated the ways in which this knowledge could be employed for anti-British propaganda to Nazi Germany’s advantage. It is noteworthy that *Indien* did not betray any traces of the contempt displayed by Hitler and a few of his associates towards Indians (Voigt 1971: 33–65). In his representation of India’s ancient past, Alsdorf dutifully gave the nod towards the “Aryan race theory” as it was applied to India by ethnologists and anthropologists who found favour with the NS regime, like Hans F.K. Günther and Egon Freiherr von Eickstedt (Alsdorf 1940: 233). But he refrained from commenting on the purported racial deficits ascribed to Indians by a strand of German Indology, spearheaded by the Indologist Christian Lassen’s seminal *Indische Altertumskunde* (Indian Archaeology, 4 vols., 1843–1862). This influential work propounded a binary narrative of fair skinned Aryans invading India and subjugating the dark skinned aboriginals, who were allegedly physically and intellectually inferior (Roy 2016: 218).

The political message of *Indien* was in tune with Nazi propagandistic overtures towards various British-ruled countries, deploying anti-British nationalism and indigenous insecurities. Alsdorf used such tropes by denouncing the British in India due to their “unrestrained brutality and cruelty which has also shocked us in Ireland and Palestine” (Alsdorf 1940: 61). Alsdorf justified Nazi racial politics while castigating British colonialists as racist. He claimed that although it was correct to maintain a separation between Indian and British “races,” it was not fair to deny even upper class Indians entry into exclusive British establishments, since, unlike the insignificant Jewish minority in Germany who did not belong to the country, Indians were natives of India. Alsdorf also accused the British of engendering a racial inferiority complex among Indians and then cleverly exploiting it after the “German revolution of 1933” to generate anti-German feelings among Indians (Alsdorf 1940: 64). Notably, Alsdorf praised Gandhi for bringing the “silent masses” of Indians into the fold of the Indian nationalist movement under the Indian National Congress or INC (Alsdorf 1940: 180). This was a deviation from Rosenberg’s contention that racial degeneration of Indians has produced only “the tired Gandhi” (Rosenberg 1934: 662) or Göring’s view that Gandhi was a Bolshevik (Voigt 1971: 37). Alsdorf’s portrayal of Gandhi reinforced Nazi Germany’s policy under Ribbentrop which entailed expressing support for the Mahatma.

Alsdorf’s depiction of Gandhi also exhibited certain characteristics of German Romantic Indology. He imparted on the Indian political leader the spiritual aura of a Hindu yogi, for whom politics and religion coalesced in two principles, truth and non-violence. For Alsdorf, Gandhi’s propagation of the ethics of *satyagraha *(insistence on truth) symbolized a return to the “original Indian spirit” found in the oldest religion of the Indo-Iranian Aryan peoples (Alsdorf 1940: 170–171). Along with “Orientalising” Gandhi and “Aryanizing” *satyagraha*, Alsdorf cast doubts on the efficacy of the Mahatma’s non-violent methods. He claimed that Gandhi’s passive resistance was easier for the British to deal with than radical and violent politics (Alsdorf 1940: 229). Alsdorf concluded that *satyagraha*, however noble it was as a moral philosophy, was not equipped to win independence for India (Alsdorf 1940: 230). He also propagated another Nazi tenet, dictatorship, by claiming that parliamentary democracy was unsuitable for India (Alsdorf 1940: 220–221). Alsdorf ended the book with a prophecy that this war could be of “revolutionary significance” (*umwälzender Bedeutung*) for India, thereby hinting that Indians should use this opportunity to end colonial rule (Alsdorf 1940: 230).

*Indien* was deemed to be of “exceptional political importance” by the Nazi party and its second edition was prepared at the “Führer’s personal interest.”^34^ The success of the book ended all doubts about Alsdorf’s commitment to Nazism with a finality that his manifold political gestures had failed to attain. More importantly, it demonstrated his “political usefulness” for the “Third Reich.” Conversely, Alsdorf’s position as a member of the academic elite who possessed first-hand knowledge of India imparted an unique intellectual authority to the book. This episode reveals that, to the Nazi political establishment, the value of a scholar was dependent on his (it was rarely hers) perceived usefulness in deploying his knowledge to answer “practical” issues faced by the regime. This “useful knowledge” had to be related to the scholar’s area of academic specialization but the two categories of knowledge did not have to be identical.

The extent to which *Indien *established Alsdorf as an authority on modern India, particularly with regard to British colonization, is evident from the fact that soon after its publication two competing political authorities enlisted his services. Alsdorf wrote a memorandum entitled “On the exercise of British domination on India”, which Alfred Rosenberg, then Minister for the Occupied Eastern territories, presented to Hitler in autumn 1941. Hitler supposedly described the memorandum as “very interesting” (Voigt 1971: 50). This prompted Rosenberg’s rival Ribbentrop to submit a “counter report” entitled “Foundation, development and methods of British domination in India” which was composed primarily by Alsdorf (Kuhlmann 2003: 165).

## Special Office India

In April 1941, the militant Indian anti-colonialist politician Subhas Chandra Bose (1897–1945) arrived in Berlin as a political exile, having escaped house arrest in Calcutta. Bose wanted to form an alliance with the Axis powers in order to secure India’s independence through military means (Kuhlmann 2003: 140). His goal was to convince Nazi Germany to declare India’s independence and allow him to form a government of free India in exile. The aim of the Nazi regime was to use Bose as a propagandistic symbol of Germany’s sympathy for India’s anti-colonial movement (Roy 2022b: 2). It is, however, imperative to note here that the Nazi government was not the first to instrumentalize the nationalist aspirations of Indians and the relevant knowledge of German academics specializing in India. As various historians have noted, the German Foreign Ministry supported the formation of an Indian Independence Committee, comprising a group of anti-colonial Indians in Berlin in 1914. The committee conducted anti-British propaganda as well as other activities (Barooah 2004; Liebau 2014; among others). The German Foreign Ministry’s efforts to engage in anti-British propaganda with the help of the Indian diaspora involved the young Indologist Helmuth von Glasenapp whose collaboration with the German government foreshadowed that of Alsdorf. Glasenapp was, like Alsdorf, an expert on Jainism (McGetchin 2010: 105). However, unlike Glasenapp, whose political engagements were limited primarily to the years of the First World War, Alsdorf’s deployment of certain kinds of knowledge of India was consistent with his deep involvement in various aspects of Nazi politics, as we shall see in the following section.

Hitler refused to declare India’s independence. Nevertheless, Ribbentrop set up a *Sonderreferat Indien* (Special Office India, henceforth SRI) out of the “Working group India” which had existed within the Foreign Ministry since 1940 to carry out the regime’s policy towards India. This policy involved mostly print and radio propaganda (Kuhlmann 2003: 158). The SRI was placed under Wilhelm Keppler, state secretary for special duties and a high-ranking SS functionary. The working director of the SRI was Adam von Trott zu Solz, an Oxford educated aristocrat who was secretly working against Hitler (Barooah & Barooah 2015: 86–88). In May 1941, Ludwig Alsdorf was appointed as an “academic assistant” at the SRI, for which he was given a lien from the University of Münster and exempted from the war duties to which he had been summoned on 3 April 1941.^35^

Subhas Bose was allowed to head a “Free India Centre” (henceforth FIC) in Berlin. This apparently independent organization comprised Indian men living in Germany and France (Kuhlmann 2003: 166). The SRI was to “supervise” the activities of the FIC, which carried out radio and print propaganda championing the cause of India’s independence, Axis victory and Bose’s activities.^36^ The other duties of the SRI entailed the collection of information relating to India and the production of different kinds of propaganda concerning India. According to a statement given by A.C.N. Nambiar, the journalist and anti-colonialist who became Bose’s deputy at the FIC, to British counter espionage agents in Switzerland in 1947, Alsdorf acted as an intermediary between the SRI, the FIC and the Foreign Ministry.^37^ Alsdorf deployed his “expert knowledge” for the SRI in multiple ways, the first and most voluminous of which was a series of fortnightly reports on India. Between 1941 and 1944, the SRI provided Ribbentrop and other important functionaries of the Foreign Ministry with regular reports on political, economic and military/strategic developments in India. Many of these reports bear Alsdorf’s signature or initials, indicating his authorship. Others exhibit his signatures alongside that of his colleague at the SRI, Franz Josef Furtwängler, an erstwhile Social Democrat and labour union leader. Furtwängler had visited India and had written works about the country, earning himself a reputation as a non-academic “practical expert” on India (Barooah & Barooah 2015).^38^ These memoranda contributed to Germany’s policymaking towards India, as manifested in the news and opinions expressed by media outlets in Germany and the territories controlled by it. Nambiar claimed that Hilmar Baßler, the Secretary of the Press department of the Foreign Ministry who was in charge of the affairs of the “Far East,” was guided by Alsdorf in deciding what was to be published on India.^39^

The memoranda written by Alsdorf provide an idea of which aspects of contemporary India were considered “politically significant” by the German government. Particularly important were the opinions and activities of the leaders of the INC. Apart from Gandhi, whose personality and political ideology towered over the party even after he formally renounced his membership, Alsdorf regularly reported on other prominent Congress politicians as well. Alsdorf kept watch across the political spectrum on both the left and the right of the centrist faction that dominated the INC. He wrote about the activities of the Indian Communist Party as well as the “Congress Socialists,” the left-wing section of the INC. He also tried to keep an eye on the radical revolutionaries who resorted to violence as part of their anti-colonial agenda. Borrowing the terminology used by the British colonial authority, Alsdorf referred to such revolutionary anti-colonialism as “terrorism.” Right-wing politics, particularly the emergent Hindu nationalist movement, were also keenly observed by Alsdorf. The response of the British administration in London to the Indian anti-colonial activities and demands formed another subject of his reports. He also gave considerable importance to the impact of the war on India as well as the geopolitical importance of India in the war. He and Furtwängler often provided exaggerated accounts of India’s anti-British unrest. Alsdorf’s primary concern in writing about the developments in India was to determine their usefulness for German propaganda.

Among the memoranda written by Alsdorf, particularly significant were those concerned with the greatest dilemma facing the German Foreign Ministry: how to react to the burgeoning movement for Pakistan, a separate homeland for Indian Muslims that was to be carved out of the Indian subcontinent. The INC, led by Gandhi and Jawaharlal Nehru, who advocated a secular and pluralist India, was against the idea of Pakistan, while the separatist party, the All India Muslim League, demanded it. Bose, who endorsed a united anti-colonial struggle of all Indians irrespective of their faiths, was also against this separatist movement. He thus insisted that German propaganda in India should desist from using religion based rhetoric or imagery and from attaching any importance to the Pakistan movement (Roy 2022: 6).

This posed a problem for the Foreign Ministry, which had already embarked on a wartime strategy of courting Muslim support worldwide, for which Islamist rhetoric was often employed (Herf 2009; Motadel 2017). Alsdorf addressed this subject in a number of memoranda. An important memorandum written by Alsdorf to address this problem was titled “The Indian Muslims, the Pakistan plan and the German politics of the Orient,” dated 2 December 1941. In this note, Alsdorf stressed the importance of cultivating the support of India’s 90 million Muslims who, due to their sheer number, were important for Germany’s entire central Asian and Near Eastern strategy. Alsdorf suggested that the subject be considered an internal matter of India which was not to be commented on.^40^ Keppler presented this memo to the Foreign Ministry on behalf of the SRI.^41^ It possibly contributed to Ribbentrop’s directive from February 1942 that propaganda for India should not contain Islamic religious rhetoric.^42^ Alsdorf returned to this subject in May 1943, after Bose had left Germany for East Asia. Certain functionaries of the Foreign Ministry like Wilhelm Melchers of the “Orient Office” who was responsible for “Orient propaganda,” had begun to claim that keeping silent on the Pakistan movement would alienate the Arab countries which sympathized with the separatist aspirations of the Indian Muslims.^43^ Keppler however wanted to continue Bose’s policy. To assist Keppler, Alsdorf wrote a number of memoranda on Indian Muslims in which he reinforced the necessity of opposing the Pakistan Plan. In a report on Muslim organizations in India, Alsdorf conceded that the Muslim League was indeed the strongest and most active of all the Islamic political organizations in India, but he managed to establish that the League was the only one among them to demand a separate state for Indian Muslims.^44^ Another memorandum, dated 6 May 1943, underscored the necessity of addressing this topic in the German media. It advocated that German propaganda should chide the followers of the Muslim League for being manipulated by the British.^45^ In another memorandum, dated 15 May 1943, Alsdorf claimed that most Muslims and practically all Hindus of India were against the idea of Pakistan. They would remain grateful to Nazi Germany for being on their side.^46^ Alsdorf also expressed similar ideas in an undated tract, presumably written around the same time, entitled “Pakistan, the Indian Ulster” wherein he compared the Indian situation to that of Ireland and Palestine and portrayed Muslims as the victims, following the tune of Nazi propaganda.^47^ Ultimately, a compromise was reached between the SRI and the Orient office. At a special meeting of the “India Committee,” comprising members from both organizations, convened on 20 May 1943, it was decided that German propaganda would not directly criticize the Muslim League and the Pakistan movement to avoid hurting Islamic sentiments. At the same time, no separatist movements would be openly supported.^48^ Alsdorf thus played a role in determining Nazi Germany’s official stance towards a particularly sensitive and crucial issue pertaining to colonial India.

Alsdorf’s attitude was guided solely by the demands of German politics. Personally, he seemed to have inherited an anti-Islam bias that was extant both in the Indological tradition that engaged with Vedic Aryanism and in the British colonial historiography (Roy 2016: 219–220). Alsdorf’s prejudices were reflected in his occasional references in *Indien* to “Mohammedan fanaticism” and the “Turkish brutality” of Indian Muslims, to whom he also attributed “low intellectual capacity” (Alsdorf 1940: 88). According to Nambiar’s post-war testimony, Alsdorf acted as the liaison between the SRI and Jamil Ahmad Khan, an Indian soldier turned Axis ally who was responsible for Italian propaganda concerning India.^49^ Italian propaganda was more inclined to employ Islamic religious rhetoric and to support the Pakistan movement.^50^ There is no record of Alsdorf opposing such pro-Islamist propaganda.

In a report written in October 1944, Alsdorf commented that it was beneficial for German propaganda that Gandhi continued to oppose the Pakistan plan, which was supported by the British. If an agreement on this subject had been reached, there would have possibly been a compromise between the Indians and the British, which was detrimental to Germany’s interests.^51^ On this issue, Alsdorf used his knowledge pragmatically, proving once more his usefulness to Nazi politics.

Alsdorf’s duties at the SRI were not limited to the theoretical. He was involved in “Operation Tiger,” an ambitious and ultimately unsuccessful plan of the *Abwehr*, the secret service of the *Wehrmacht* (Armed Forces), to incite a major anti-British uprising among the tribes in India’s North West Frontier Province (Hauner 1981: 235–236). Bose was invited to take part in planning this operation, which had a precedent in a plan conceived during the First World War by the German Foreign Ministry and Indian anti-colonialists in Berlin (Stewart 2014). In a meeting held on 11 August 1941, in which Bose and spokesmen from the *Wehrmacht* and the *Abwehr* were present, Alsdorf and Trott represented the SRI.^52^ In addition, Alsdorf occasionally participated directly in the transfer of information between the German Legation in Kabul and the Foreign Ministry in Berlin. In 1944 (if not earlier) Alsdorf sent telegrams with coded messages to the Legation of Kabul. Sometimes he acted as a conduit between Bose, who was then in East Asia, and his agent in the Indian subcontinent, Bhagat Ram Talwar (who used the pseudonym Rahmat Khan or RK) by sending as well as receiving coded messages through Kabul.^53^ Another of Alsdorf’s “practical” engagements was to act as a liaison between the Foreign Ministry and the Indian Legion or Infantry Regiment 950, a joint venture between Bose, the Foreign Ministry and the *Wehrmacht*. All the soldiers in this regiment were recruited from the Indian POWs who had fought the Axis powers in North Africa as part of the British Army (Rose 1979; Hartog 1991). Gurbachan Singh Mangat, one of the soldiers who joined this Legion, recounted in his memoirs how the Indian soldiers were once welcomed at a Berlin restaurant by Alsdorf, who wore the uniform of a Lance Corporal and spoke in refined, accent-free Hindustani. Subsequently, Alsdorf introduced the solders to Trott and others functionaries of the Foreign Ministry (Mangat 1986: 60–61).

## Public Propaganda

Alsdorf not only influenced the German media from the wings, but he also wrote propagandistic texts on India for general readers. Noteworthy among such writings was a polemical pamphlet entitled “India’s way to freedom” which Alsdorf wrote under the pseudonym Botho Ludwig. It was published in 1942 by the Berlin-based Walter Titz Verlag, known for publishing propagandistic tracts relating to foreign affairs (Hensel 2019). In this booklet, Alsdorf traced the development of the Indian anti-colonial movement. He praised Gandhi, but focussed more on Bose, whom he portrayed as a forward thinking man of action with an unparalleled nationalistic fervour. The book ended by prophesying the victory of the Indian anticolonial movement and proclaiming, somewhat ironically for someone working for the Nazi government, “no one can sit on bayonets, especially when the bayonets are required all over the world” (Ludwig 1942: 64). Similarly polemical was an article that Alsdorf wrote for the *Berliner Börsen Zeitung*, entitled “Indien kämpft” (India fights), published on 25 August 1942. It claimed that the Indians, following the imprisonment of their “Führer” Mahatma Gandhi whom they revered like a god, had embarked on a grim battle against the British Empire, which now lay fatally weakened (Alsdorf 1942a).^54^

## Free India Centre

According to Mukund Rai Vyas, Bose’s secretary and a leading functionary of the FIC, after Trott was executed in connection with the attempt on Hitler’s life on 20 July 1944, Alsdorf became the de facto head of the SRI.^55^ This statement was given by Vyas to the British authorities at Brunswick prison in June 1945 (Vyas 1982: 520). Nambiar also claimed in his statement from 1947 that after July 1944 Alsdorf became Keppler’s principal deputy. Alsdorf was “best informed about the FIC and about communications with Bose in the Far East” but the Indologist was not particularly forthcoming with the information as far as the Indians were concerned.^56^ As the liaison between the SRI and the FIC, Alsdorf visited the FIC’s office several times after it moved to Hilversum in Holland in October 1943 to avoid Allied bombing.^57^ His last visit to the FIC seems to have taken place in early 1945. By that time, the FIC had moved again to Helmstadt in Bavaria. On this visit, Alsdorf circulated pamphlets in English, Hindustani and Urdu, to be distributed among the Indian soldiers who were fighting on the continent as part of the British Army. The pamphlets, designed by the propaganda group under the aforementioned Jamil Ahmed Khan in Italy, asked Indian soldiers to desert their colonial masters and to join the Axis cause.^58^ Alsdorf thus pursued the political objectives of Nazi Germany until the bitter end.

## Book Series on India

A book series titled “India through individual portrayals,” was SRI’s project to generate written materials in German “that would provide a clear source of information relevant to external politics for all the organisations that had something to do with India,” as Trott claimed in a note on 23 January 1942 (Kuhlmann 2003: 163). The books were also meant to demonstrate to the world that Germans were genuinely interested in India, as Alsdorf wrote in an official notice.^59^ As a sign of German Indian co-operation, the series was to comprise eight books, written by four Indians and four Germans. Furtwängler was named as the series editor. Kurt Vowinckel, the Heidelberg based right-wing publisher, was to publish the series comprising the following titles: Hermann Beythan: What is India (*Was ist Indien*); Hermann Lufft: The Indian Economy (*Die Wirtschaft Indiens*); Koodavuru Anantrama Bhatta: India in the British Empire (*Indien im Britischen Weltreich*); Mukund Rai Vyas: Men and Political Parties in Today’s India (*Männer und Mächte in heutigen Indien*); Abid Hassan: Islam in India (*Islam in Indien*); Abdul Quddus Faroqhi: The Social Question in India: (*Die Soziale Frage in Indien*); Ludwig Alsdorf: The Spiritual Connections between Germany and India (*Deutsch-indische Geistesbeziehungen*); Wilhelm Kruse: Monuments in Indian Art (*Denkmaler Indischer Kunst*) (Kuhlmann 2003: 162).

The venture soon ran into troubled waters as the manuscripts by the Indian authors were not considered worthy of publication by Furtwängler, who was “acting on the advice of Prof Alsdorf.”^60^ The latter “corrected” three of the manuscripts, which were then published under the names of Bhatta, Vyas and Hassan, who complained to Bose that drastic changes had been made to their manuscripts without their knowledge or consent.^61^ Bose in turn complained to Keppler, as a result of which “the fiction of the principle of parity” (in the words of Furtwängler and Alsdorf) was finally abandoned. Faroqhi’s manuscript was returned unused.^62^ The published book bore the name of the scholar Hermann Beythan, who actually wrote it. Alsdorf also took a leading role in rejecting the manuscript on Gandhi by Devendra Nath Bannerjea, an Indian intellectual living in Berlin who enjoyed the trust of several powerful functionaries in the German government. Subsequently, Alsdorf defended Furtwängler, when Bannerjea, presumably as an act of revenge, tried to denounce the erstwhile leftist trade union leader for allegedly conspiring against Hitler.^63^

In this context, it is pertinent to mention that Alsdorf also had a role in preventing the publication of the German translation of Subhas Bose’s book, *The Indian Struggle*. Bose was eager to publish the work, for which he had secured the approval of the Foreign Ministry. In an official note to Keppler, dated 29 September 1942, Alsdorf claimed that the exhaustive political details of the book would tire German readers. Moreover, “politically the book was a continuous apologetic-polemical debate with Gandhi, whose mistakes at every step were reviewed.” The publication of such criticisms, claimed Alsdorf, would go against Germany’s political stance towards India, which carefully avoided any approach that could be interpreted as disparaging to the Mahatma or as an assault against him.^64^ According to Vyas, Alsdorf took it upon himself to make a round of changes to the draft in 1944. However, it was never published.^65^ This episode points to the influence that Alsdorf had amassed at the SRI, indicating the Nazi regime’s increasing trust in him.

The book that Alsdorf wrote for this series, “The Spiritual Connections between Germany and India” (*Deutsch-indische Geistesbeziehungen*) had an unique propagandistic value, as Erxleben from the Nazi party’s “department of Sciences” (*Wissenschaft*) claimed in his report dated 22 May 1942.^66^ The propagandistic mission of the book is manifested in Alsdorf’s claim that a sense of solidarity prevailed between colonized Indians and Germans who chafed under the unjust treaty of Versailles until 1933 (Alsdorf 1942b: 1–2). The limits of this solidarity, however, became evident soon enough, through Alsdorf’s hegemonic contention that “just as the people from other nations need to learn German to enjoy the fruits of German research on medicine, science and technology, Indians need to learn German in order to fully participate in researching their own history and culture, religion, philosophy, literature and language” (Alsdorf 1942b: 3). The argument posited by the book was that while the British colonized India and siphoned off its material wealth, Germans were interested only in unearthing and preserving the intellectual treasures of India, which they did with great success (Alsdorf 1942b: 3). This book was a paean to German Indology, the evolution of which was lucidly traced by Alsdorf in order to make the Nazi state aware of the contribution of German Indologists to the nationalist project of enhancing the prestige of German academia in the world.

## *Aktion Ritterbusch*

A similar effort by Alsdorf to highlight the achievements of German Indology was evident in 1942, through his contribution to the *Aktion Ritterbusch*. This “action” represented the so-called “war efforts” of the humanities in German universities between 1940 and 1945, under the direction of the legal scholar Paul Ritterbusch. This enterprise entailed a battle waged with words, comprising papers and monographs published by scholars with the aim of demonstrating the superiority of the “German spirit” and the German “sciences” (*Wissenschaften*) over “western” (mainly French and British) thinking (Hausmann 2011: 84). As part of this project, a conference of German orientalists was organized in Berlin from 30 September to 3 October 1942. Here, Alsdorf presented a paper titled “The Indian freedom movement” (*Die Indische Freiheitsbewegung*) which was later published in a book edited by the Orientalist Hans Heinrich Schaeder in 1944. In this paper, Alsdorf emphasized the supposedly unique position of German Indologists as experts capable of understanding and explaining the Indian anti-colonial movement to the Germans (Alsdorf 1944: 217, 226). Alsdorf thus once again advertised the indispensability of German Indologists in the venture of deploying the Indian anti-colonial movement for Nazi aims.

## Chair for Indian Studies

Alsdorf’s meteoric ascension to the firmament of Nazi academic politics was evident by 1942, when he was considered for two professorial posts—one at Alfred Rosenberg’s institute, the *Hohe Schule* in Munich, and the other at the faculty for the Study of Foreign Countries (*Auslandswissenschaftliche Fakultät* or AF), which was founded in 1940 and affiliated to the University of Berlin. Alsdorf was ultimately “awarded” to the AF.^67^ Securing Alsdorf for his *Hohe Schule* was so important to Rosenberg that he wrote an official letter on the subject to Martin Bormann, the powerful head of the Nazi party’s chancellery in Berlin. In this letter, Rosenberg claimed that Alsdorf’s real strength as an academic was in the sphere of the “great and ancient India,” an area of research that was urgently required for ideological reasons.^68^ However, the Foreign Ministry, the Education Ministry and the SS, which worked together to establish the AF, decided that Alsdorf’s expertise on modern India was politically more valuable. This contest between the merits of the knowledge of ancient India for legitimizing Nazi ideology versus the ability to provide insights into modern India for the sustenance of German Realpolitik represented the different possibilities of the “application” of “expert knowledge” of India in Nazi Germany.

Certain demands of Nazi foreign policy and the realities of war led to the foundation of the AF and along with it, the *Deutsche Auslandswissenschaftliches Institut * also affiliated to the University of Berlin. While the AF focussed on teaching, the DAWI concentrated on research and publications. Both institutes pursued knowledge that was considered “politically useful” (Botsch 2006). The dean of the AF as well as the director of the DAWI was Franz Alfred Six, professor of print media studies (*Zeitungswissenschaft*) and a member of both the SS and the SD. In 1943, Six became the head of the “cultural political department” which included the SRI, at the Foreign Ministry (Hachmeister 1998: 244).

Even before the SRI came under his sphere of authority, Six was keen to have a professorship devoted exclusively to modern India at the AF, for which Alsdorf appeared to be the most eligible candidate. As Six wrote to the Education Ministry on 16 January 1942, Germany would have to deal directly with an independent India after the imminent dissolution of the British Empire. It was therefore important for Germans to have the political knowledge required to conduct this bilateral relationship. Ludwig Alsdorf was the only viable professorial candidate, since he had already proved his expertise on contemporary India, particularly through his book, *Indien*.^69^

Six had his way and in March 1942, Alsdorf was instructed by the Education Ministry to hold lectures and courses on the “People and Land of India” at the AF until the chair was officially established.^70^ He was made an “*Außerplanmäßger Professor*” (“extraordinary professor,” a post akin to lecturer).^71^ The Nazi Lecturers’ Association (NSDB) supported this step, as an evaluation from February 1942 shows. There was no trace of the NSDB’s earlier reservations about Alsdorf in this report, which claimed that he was politically “completely unobjectionable”^72^ This is another indication that Alsdorf had by then established his Nazi credentials. Notably, in a report written in November 1941, the dean of the Philosophical Faculty at the University of Münster supported Alsdorf’s promotion to “*Außerplanmäßger Professor*” claiming that Alsdorf’s “special merit” was his knowledge of modern India, accrued during his long stay there, which helped him to write his “extraordinary” book, *Indien*. The report made it clear that Alsdorf’s knowledge of modern India was far more advantageous for his career than his academic training relating to Jain literature, which the dean mentioned only in passing.^73^ Alsdorf was initially not too eager to join the AF. By the middle of 1942, he was lobbying for the establishment of an “India Institute” under the aegis of the Foreign Ministry. He claimed that such an institute would “supplement and promote the political work of the Foreign Ministry concerning India, through academic research.” The institute should be closely connected to the SRI, “if possible through a personal union.” Presumably, Alsdorf envisaged himself as the person symbolizing this “union.” Echoing the arguments expressed by Six, Alsdorf wrote that such an institute was “an urgent necessity” for the period after the war. Moreover, such an institute would be good for propagating Germany’s benevolence towards India.^74^

This wish was not fulfilled due to Six’s political clout. In 1943, the department of “People and Area Studies of India” was officially established at the AF, where Alsdorf lectured on *Indische Realien* or the “realities” of contemporary India. In April 1944, he was made *Außerordentlicher Professor*, a post akin to a reader or an associate professor. He was simultaneously made the head of the Research section on India at the DAWI (Botsch 2006: 298). This episode makes it clear that without the nimbus of the SS or a similarly important political authority, a scholar’s influence, even if he proved his practical use to Nazi politics, had its limitations. Alsdorf held lectures at the AF until the winter semester of 1943–1944, when he, along with many functionaries of the Foreign Ministry, shifted to Krummhübel (in present day Czech Republic) to escape Allied bombings (Botsch 2006: 299).

## DAWI

The DAWI conducted “politically useful” research that found its way into propagandistic publications, which included a series of books titled *Kleine Auslandskunde *(“Brief accounts of foreign countries”). Officially edited by Six, the series provided readers “practical” with information about different countries in slim volumes and in an accessible style (Botsch 2006: 146). The cover of each book had a blurb pompously announcing that the books were written from the viewpoint that Germany’s newly achieved position as a world power required a deeper understanding of foreign countries. Ludwig Alsdorf contributed a book to this series in 1943. In this book, entitled *Indien und Ceylon*, he reinforced the propaganda line espoused by Bose and Keppler by portraying the INC as the “real” representative of Indian Muslims, unlike the separatist Muslim League (Alsdorf 1943a: 46). In the book, Alsdorf portrayed the history of Ceylon (Sri Lanka) through the prism of German Indology, claiming that the island was “conquered” by Aryans from north India (Alsdorf 1943a: 47). He described Sri Lanka as a more blatant example than India of a “tropical colony for exploitation” (Alsdorf 1943a: 142). Alsdorf also regularly wrote articles on India for the DAWI, which published a monthly journal, a yearbook and several periodicals. A significant “report” by Alsdorf in the 1942 Yearbook was on the “Führer’s” reception of the “radical Indian nationalist” and the “leader of Bengali activists” S.C. Bose on 27 May 1942. Alsdorf presented this meeting as a symbol of Germany and India’s joint struggle against Britain (Alsdorf 1943b: 643–644). In reality, the meeting was patently disappointing for the Indian leader (Kuhlmann 2003: 228–229).

## After the war

The DAWI and the AF were dissolved soon after the war, leaving Alsdorf without a job. His obituary mentions that, from 1945 to 1948, Alsdorf lived with relatives in the countryside of Rhineland Palatinate (Bruhn 1990: 7). The obituary goes on to state that in 1948 Alsdorf was able to return to his former workplace, the University of Münster, as a guest professor. The missing details about these years provide crucial insights into Alsdorf’s attempts at coping with his tainted past. Alsdorf’s membership in the NSDAP meant that he had to go through the legalities of denazification, during which he was indicted as a *Mitläufer* (“fellow traveller”), belonging to Category IV of political culpability.^75^ This presented a hurdle to entering post-war German academia. Although he was given permission to teach at the University of Münster by the military government in June 1947, the Ministry of Education and Culture of the state of North Rhine Westphalia (NRW) was reluctant to give the required directive. Alsdorf’s “political liability” led the University of Munich to reject him as a possible professorial candidate in 1948.^76^ The University of Münster however was interested in Alsdorf’s return to the practically defunct department of Indology. To bypass the impediments brought about by Alsdorf’s proven complicity with the Nazi regime, University authorities, Alsdorf himself and a number of scholars embarked on a remarkable political strategy.

The classicist Franz Beckmann, dean of the Philosophical Faculty at the University of Münster, officially requested that the Ministry of Education and Culture approve Alsdorf’s appointment as a guest lecturer at the “currently orphaned” department of Indology.^77^ Beckmann’s sympathy for Alsdorf is not surprising, since he was also associated with Nazi politics. He was in charge of the “cultural supervision” of the German Army in the Balkans from 1943 onwards. Such “supervision” usually translated into inculcating the regime’s racist and imperialist ideals into the soldiers as a way to boost their fighting morale (Baranowski 2004: 207). After 1945, he managed to present his activities as completely apolitical and purely cultural.^78^ Beckmann’s attempts were continued by his successor to the dean’s office, the historian Herbert Grundmann, who had collaborated with the regime in various ways, which included conducting radio propaganda. Since he was not a member of the Nazi party, Grundmann was classified as “politically unencumbered” (Nagel 2004: 601–603).

On 13 April 1948, Grundmann sent a letter to the North Rhein-Westphalian Ministry of Education and Culture.^79^ This letter deserves a detailed examination since it is a striking example of retrospectively manipulating a politically compromised biographical narrative. The letter stated that Alsdorf’s entry into the NSDAP and the NSKK were due to his justified fear that, without such affiliations, he would not be able to continue his academic career. It claimed further that at the University of Berlin, Alsdorf was pressurized in this regard by Bernhard Breloer. Conveniently, Breloer had died in a Russian prison in 1947 (Losch 1955). The letter also resorted to the widespread post-war myth that the NSKK was a lesser evil compared to the SA and the SS. Grundmann’s claims were based on Alsdorf’s post-war application to the University of Munich, wherein Alsdorf stated that Breloer coerced him into joining the NSKK. Once enrolled, he could not avoid taking part in the service routine.^80^ Grundmann maintained that despite his membership in “the party” and the NSKK, Alsdorf retained his scholarly integrity, as evidenced in *Indien*, which was recognized by academics as a work of sound scholarly quality, while the Foreign Ministry and the Ministry of propaganda criticized it as “Anglophile.” The letter went on to claim that Alsdorf continued to work for the Foreign Ministry despite, and not because of, the book. Moreover, Alsdorf worked for the SRI under Adam von Trott zu Solz, one of the “victims of 20 July 1944.” This attempt to exculpate Alsdorf by associating him with Trott was similar to the post-1945 efforts of certain officials in the Foreign Ministry to use Trott as a symbol of the Ministry’s purported bid to resist Nazification. In reality, Trott’s opposition to Hitler was one of a few individual cases inside the Ministry, in which Trott remained something of an outsider (Conze et al. 2010: 12–13, 16–17).

Accompanying the letter were statements from leading Orientalists affirming Alsdorf’s lack of guilt. The signatories included Walther Schubring, Hans Heinrich Schaeder, Helmuth von Glasenapp and Franz Taeschner. The latter, it should be recalled, was a member of the NSDAP who had used his academic expertise to serve the NS state. Schubring was no opponent of the regime either. He had signed the oath to Adolf Hitler in 1933, although he did not join the Nazi party (Pollock 1993: 94). Schubring had recommended Alsdorf to the University of Münster in 1938, claiming that he was “naturally fully unobjectionable in his politics.”^81^ Schaeder conformed to certain aspects of the Nazi worldview, which he promoted in various ways, for example by editing the aforementioned book as a part of *Aktion Ritterbusch* (Schuster 2017: 686–689). After 1945, by attesting to Alsdorf’s “inner distance” from Nazi politics, these scholars were also distancing themselves from their own pasts. The “politics of history” conducted by Alsdorf and his cohort met with success on 19 May 1948, the Ministry of Education and Culture of NRW gave Alsdorf the official permission to become a guest lecturer at the University of Münster. He started work on 20 June.^82^

Interestingly, the University of Münster was so keen to reappoint Alsdorf that it refused the Ministry’s offer in December 1947 to engage Walter Ruben, the Jewish Indologist who had migrated to Turkey to avoid persecution and was contemplating a return to Germany.^83^ On 21 January 1948, Grundmann wrote to the Ministry that the respected Indologist Walter Ruben could be considered only for a full professorship, which was not available at the University of Münster.^84^ This answer was based on a “report” provided by Alsdorf himself, who had been asked to comment on the situation by Grundmann.^85^ If this episode denotes a continuity with the years of Nazi rule, the projection of Indology’s “usefulness” underwent a transformation after 1945. A letter, written by Walther Schubring in April 1948 to the Faculty of Philosophy at the University of Münster, claimed that Indology had contributed greatly to the prestige of German *Wissenschaft*. Alsdorf’s reappointment would enable him to bring in new honours for the discipline. Moreover, through Alsdorf’s personal contacts with Indian scholars, Münster could be a source of profitable academic relationships with India.^86^ Claims of “national prestige” and “international connections” were often used by German academics and institutions after 1945 to re-establish themselves (Schüring 2006: 332).

In 1950, Alsdorf succeeded Schubring as professor of Indology at the University of Hamburg where he remained until his retirement in 1972.^87^ By the time of his death in 1978, Alsdorf had established himself as a renowned Indologist. His obituary mentions his early academic works related to Jainism and discusses his prolific academic writings after 1945 (Bruhn 1990: 5–13). His wartime writings on modern India are acknowledged only through a few innocuous lines (Bruhn 1990: 6). An editorial note in the same volume mentioned that the three books on modern India written by Alsdorf were meant to provide information to Nazi political circles. However, he “did not make any concessions to the ideology of the Third Reich (Bruhn et al. 1990: ix).” In this way, the political significance of Alsdorf’s writings on modern India was expunged.

## Ludwig Alsdorf, German Indology and Indian Anti-Colonialism

This article has examined Ludwig Alsdorf’s role as a scholar serving Nazi political goals, for which he received professional advancement. It has also studied the complicated relationship between institutional and “practical” knowledge and the Nazi state as far as the academic discipline of Indology is concerned. Alsdorf deployed his knowledge of India to fulfil a set of cultural political aims of the Nazi government. It was this political “usability,” established through his book *Indien* rather than his membership in the Nazi party and its affiliates, which brought Alsdorf professional opportunities and influence, particularly at the Special Office India. By contributing his knowledge of India to further the interests of Nazi cultural politics, Alsdorf fulfilled the Nazi regime’s primary expectation for scholars and academics. At the same time, Alsdorf’s academic credentials validated the “usable knowledge” that he generated, even though it differed from his area of academic specialization. After 1945, Alsdorf’s collusion with the Nazi regime and the political uses of his knowledge of India were trivialized to refashion an image of a brilliant scholar who was forced by the sinister Nazi regime to make certain compromises, which were, however, minimal. Finally, the article has analysed the mutability of the perceived uses of Indology for the “Third Reich” and the Federal Republic of Germany. The issue of the “usefulness” of Indian Studies provided the necessary backdrop for the connections between Alsdorf’s “practical” and scholarly knowledge relating to India and the different political systems.

## Footnotes

1 Biographical information on Alsdorf is based on his own 1941 account. University Archive Münster (UAM): *Lebenslauf* Alsdorf. Bestand 63, Nummer 7 Bd.1. His file from the University of Berlin claims that he joined the NSDAP in May 1933. Humboldt University Archives (HUA), Personalia Alsdorf. A 051.

2 Geheimes Staatsarchiv Preußischer Kulturbesitz (GstAPK) Berlin: IHA Rep.76 Kultusministerium. Musterbeurteilung.

3 GstAPK: IHA Rep76 Sek.11. Nr. 7a.

4 UAM: Bestand 63, Nummer. 7 Bd.1.

5 Archive of the Institut für Zeitgeschichte (IfZ) Munich (AIFZ Munich):116–1.

6 HUA: UK Personalia. Alsdorf, A051 Fragebogen.

7 UAM: Lebenslauf. Bestand 63, Number 7 Bd.1.

8 Federal Archives (*Bundesarchiv* or BA) Berlin. R4901/24118. 0594.

9 BA Berlin. R4901/24118. 0617. *Dienstleistungszeugnis*, 24.9.34.

10 BA Berlin. R4901/24118. 0628.

11 “Politisch undurchsichtig, seiner Haltung nach aber kaum als Nationalsozialist anzusprechen, auch wissenschaftlich nicht. Aufgeblasen schwätzerhaft. Sehr stark egozentrisch bestimmt.” BA Berlin. R4901/24118. 0619.

12 “… es scheint mir nicht wahrscheinlich, dass er als Nationalsozialist anzusprechen ist.” BA Berlin: R4901/24118. 0624.

13 BA Berlin: R4901/24118. 0631. Breloer to the Dean of the Philosophical Faculty, University of Berlin, 27.2.1936.

14 BA Berlin. R9361\II 10407. 060.Letter dated 14.4.1938.

15 “Im Fachgebiet seiner Wissenschaft hat er sich zu weltanschaulichen Problemen noch nicht geäußert.” BA Berlin. R9361\II 10407. 066. Breloer’s report of 3.4.1938.

16 GstAPK: IHA Rep76 Sek.11. Nr. 7a. Fragebogen.

17 HUA Berlin: NS-Doz.1, Nr. XXX. No.6. Letter dated 4.4.36.

18 18 Sächsische Hauptsstaatsarchiv (SaHStA) Dresden: 11125 Ministerium des Kultus und öffentlichen Unterrichts, Nr. 10230/57. Besetzung des Ordentlichen Lehrstuhls für indische Philologie, Nachfolge Hertel durch Weller, 1936–1938. Among the reports are those by the *Dozentenschaft* of the University of Leipzig and by Werner Studentkowski, representative of the NSDAP at the University of the Leipzig.

19 SaHStA Dresden: Ministerium für Volksbildung, 10230. Abtlg: 1b.L8.VIII.No.73.Bd.3 Heft2. 6.2.1937.

20 “Wenn er auch Parteigenosse ist, so halt er sich an der Universität meist nicht zu den Nationalsozialisten sondern mehr zu den liberalen Kreisen. Charakterlich kommt er wohl der Typ eines Strebers nahe, der alle Situationen möglichst für sich auszunutzen versucht.” SaHStA Dresden: Ministerium für Volksbildung, 10230. Abtlg: 1b.L8.VIII.No.73. Bd.3 Heft2. Letter dated 23.3.1937.

21 “Man bezeichnet ihn als nicht erfreulichen Dozententypus, der die politischen Aufgaben der Universität nicht verstanden hat.” BA Berlin. R9361\II 10407. Report dated 24.3.1938.

22 UAM: Bestand 63, Bd.2. Letter from the Education Ministry to Alsdorf, dated 31.8.1938.

23 UAM: Bestand 63, Bd.2.Letter from the Dean to the Education Ministry, dated 6.3.1938.

24 “Ich halte es für dringend notwendig, dass die arische Philologie der Universität Münster wieder vertreten wird. Ernsthafte Sprachwissenschaftliche Forschung ist ohne Eindringen in das Sanskrit nicht möglich. Es handelt sich hierbei nicht nur um rein sprachliche Fragen, sondern auch um das Problem der Urheimat der Indogermanen, das heute auch eine gewisse Nationalpolitische Bedeutung hat.” UAM: Bestand 63, Bd.2.Letter from Hoffmann, dated 9.2.1938.

25 UAM. Bestand 63, Nummer 7. Band 2. Letter from Taeschner, 15.2.1938.

26 University Archive, Halle Wittenberg: UAHW Rep. 21, Abt. III Nr. 143; UAHW, Rep. 11, PA 13985 (R. Schmidt); BA Berlin Lichterfelde BAB R 9361-VIII KARTEI / 19881207.

27 HUA Berlin: Copy of the written proof of the Hitler oath taken by Alsdorf. 8.11.1938. Bestand UK Personalia. Alsdorf, A051.

28 HUA Berlin: *Niederschrift über die Vereidigung des Dr. Phil. Habil Ludwig Alsdorf*. 19.9.1939. Bestand UK Personalia. Alsdorf, A051.

29 Bayerisches Hauptstaatsarchiv (BayHStA) Munich: MK69695. Alsdorf’s statement. P2.

30 BA Berlin: R9361\II 10407.Bl. 39. Letter dated 11.2.1940.

31 UAM: Bestand 63, Nummer 7 bd.1. Alsdorf Lebenslauf 1941.

32 UAM: *Vorlesungsverzeichnis*, Second Trimester 1940. P‑69–70.

33 AIFZ Munich: Statement by the director of the National Socialist Lecturers’ Association (*Reichsdozentenführer*) to the party office of the NSDAP, dated 18.2.1942.

34 AIFZ Munich: 116‑1. Letter to Dr Völsing, Press Office. Dated 22.4.1942.

35 UAM: Bestand 63, Nummer 7 Bd.1. On 2 April 1941, Alsdorf notified the University of Münster that he had been assigned to war duty. A letter from Heinrich Harmjanz, functionary of the REM, to the University of Münster states that Alsdorf would be working for the Foreign Ministry from 19.5.41 to 18.11.41.

36 Political Archive of German Foreign Ministry (PA-AA) Berlin. RZ 501-60671. *Areitsprogramm für die Indienpropaganda*. Undated.

37 National Archive of UK (NAUK), Kew: KV (2) 3904/2. Statement of Nambiar. P. 50 (44).

38 Copies of some of these reports are held by the BA and the PA-AA, Berlin, as well as by the NAUK.

39 NAUK: KV (2) 3904/3. Statement of Nambiar. P 58.

40 PA-AA: Handakten Keppler. R2750. *Die indische Mohammedaner, der Pakistan-Plan und die deutsche Orientpolitik*. 350492-350499.

41 PA-AA: Handakten Keppler. R27501. Keppler’s notice to various functionaries of the Foreign Ministry, dated 2.12.41.

42 PA-AA: RZ501-60670. 11.2.42.

43 BA Berlin: R901-60423. Note by Melchers, dated 7.5.1943.

44 PA-AA: RZ501-60677. *Mohammedanische Organisationen in Indien*.

45 PA-AA: RZ501-60677. *Aufzeichnung. Problem Pakistan-Moslemliga*. 6. Mai 1943.

46 BA Berlin: R901-60423. 15.5.1943. Bl.180–184.

47 BA Berlin: R901-60423. *L.Alsdorf: Pakistan, das indische Ulster*. Bl. 185–192.

48 BA Berlin: R 901/60423*. Protokoll der Sitzung des Indien-Ausschusses*, 20.5.1943. Bl.196–198.

49 NAUK: KV (2) /3904. Statement of Nambiar. P.46.

50 PA-AA: RZ501-67660. Report dated 8.3.1943. *Propagandistische Erfassung des indischen Muslims*. P4.

51 NAUK: GFM33\565. Serial number 1313. 349884. Report dated 14.10.1944.

52 NAUK: GFM33\2109. Serial No. E233480, E233481.

53 NAUK.HW12\303. Encrypted messages from Alsdorf. No. 134645 (9.8.1944). No. 135656 (18.8.1944). No.135101 (20.8.1944). No.135284 (26.8.1944).

54 National Archives of India (NAI) New Delhi. S301\43. Presseabteilung der Reichsregierung im Reichsministerium für Völksaufklärung und Propaganda

55 NAUK: KV 2/3907. Statement of Mukund Rai Vyas. Appendix D. P.1.

56 NAUK: KV2/3904/3. Nambiar’s statement. Appendix B. P.35.

57 BayHStA Munched: MK40443. Undated post-war statement of Alsdorf to the University of Munich that he went to Hilversum as a representative of the Foreign Ministry on the following dates: 20.–22.9.1943, 27.12.43–2.1.44, 2.–4.7.44, 5–6.8.44, 2.–3.9.44.

58 NAUK KV 2/3907: Statement of Mukund Rai Vyas. Appendix H1. P132–133.

59 NAUK: GFM33\565. Serial No.1313. 349809. L. Alsdorf: *Aufzeichnung*. 17.9.1942.

60 NAUK: GFM33\564. Serial number 1312. Furtwängler’s note to the SRI, 9.4.1942.

61 NAUK: GFM33\564. Serial number 1312. Bhatta’s complaint: 349823. Alsdorf to Trott zu Solz: 349815. Also, Furtwängler’s memorandum to Keppler dated 31.8.1942: 349817–349819. Undated letter by Bose to Keppler, signed as Orlando Mazzotta: Serial No.349829.

62 NAUK: GFM33\564/1312. Furtwängler’s response to “Mazzola’s complains.” 28.8.1942. Serial number 349818.

63 Relevant documents can be found in PA-AA: R27504. Handakten Keppler. Also: Maria Framke’s article in this issue.

64 PA AA: Handakten Keppler. R27504. *Aufzeichnung: Über die geplante Ausgabe von S.C.Bose’s “Indian Struggle”*

65 NAUK, Kew: KV2/ 3907. Statement of Mukund Rai Vyas. Appendix B11.

66 AIFZ Munich: MA1190.

67 AIFZ Munich: MA1190. Letter from Rosenberg’s office to NSDAP’s office, 12.6.1942. Letter from the leader of the *NS Dozentenbund* to Nazi party headquarters. 7.5.1942.

68 AIFZ Munich: MA1190. Rosenberg to Bormann, 11.5.1942.

69 AIFZ Munich: MA1190. Copy of a letter written by Six to the Ministry of Education dated 16.1.1942 and sent by the NSDAP to Rosenberg’s ministry.

70 AIFZ Munich: MA1190. Note from the NSDAP (*Partei Kanzlei*) to *Hauptamt Wissenschaft*.

71 HUA: Personal Akten Alsdorf. H51.Bd.III. Certificate from the Ministry of Education.

72 AIFZ Munich: 116‑1. Report dated 18.2.1942.

73 UAM: Bestand 63, Nummer 7 bd.1. 14.11.1941.

74 PA-AA: RZ501-60672. Alsdorf’s note to the SRI. 29.5.42.

75 UAM: Bestand 63, Bd.2. Letter from Grundmann to Alsdorf, dated 28.2.1948.

76 BayHstA Munich: MK69695. Letter from the dean of the Faculty of Philosophy, University of Munich, to the Ministry of Culture, Bavaria. 8.7.48.

77 UAM: Bestand 63, Bd.2. Letter dated 4.7.1947.

78 AIFZ Munich: ZS2178. Beckmann’s statement on 28.5.1943.

79 UAM: Bestand 63, Bd.2. Grundmann to the Ministry of Culture, NRW. 13.4.48.

80 BayHstA Munich: MK69695. Alsdorf’s post-war statement.

81 “… politisch selbstverständlich völlig einwandfrei.” UAM: Bestand 63, Bd.2. Schubring’s letter to the dean, 16.12.1937.

82 UAM: Bestand 63, Bd.2. Letter from Minister for Education and Culture, NRW to the dean of the University of Münster. 19.5.1948.

83 UAM: Bestand 63, Bd.2. Ministry of Culture, NRW to the Rector of the University of Münster, 22.12.1947.

84 UAM: Bestand 63, Bd.2. Grundmann to the Ministry of Culture, 21.1.1948.

85 UAM: Bestand 63, Bd.2. Grundmann to Alsdorf, 3.1.1948. Alsdorf’s reply, 13.1.1948.

86 UAM: Bestand 63, Bd.2. Schubring to the “Gentlemen of the Faculty of Philosophy,” 23.4.1948.

87 UAM: Bestand 63, Bd.2. Letter from the Ministry of Culture, NRW to Alsdorf, dated 14.6.1950, officially releasing him from his position at the University of Münster.
